# Extract of *Paecilomyces hepiali* mycelia induces lipolysis through PKA-mediated phosphorylation of hormone-sensitive lipase and ERK-mediated downregulation of perilipin in 3T3-L1 adipocytes

**DOI:** 10.1186/s12906-018-2389-0

**Published:** 2018-12-07

**Authors:** Mei Ge, Rui Guo, Hai-xia Lou, Wen Zhang

**Affiliations:** School of Life Sciences, East China Normal University, 500 Dongchuan Road, Shanghai, 200241 China

**Keywords:** *Paecilomyces hepiali*, 3T3-L1 adipocyte, Lipolytic effect, PKA, ERK

## Abstract

**Background:**

*Cordyceps sinensis* has been used for centuries in China as one of the most valued herbal medicine and tonic food. *Paecilomyces hepiali*, a fungal strain isolated from natural *C. sinensis,* has been used widely as a substitute of *C. sinensis* in medicine and health food. *P. hepiali* has been reported to have various pharmaceutical benefits, including triglyceride-lowing activity. However, its effects on triglyceride metabolism in adipocytes remain unknown. The purpose of the present study was to evaluate the effect of *P. hepiali* mycelia on adipocyte lipolysis and to clarify the underlying mechanisms.

**Methods:**

The fully differentiated 3T3-L1 adipocytes were treated with methanol extract of *Paecilomyces hepiali* mycelia (PHME). Contents of glycerol released into the culture medium and intracellular triglyceride were measured as indices of lipolysis using glycerol assay kit and Oil red O staining, respectively. Then, effects of PHME on the main lipases or kinases involved in lipolysis regulation were investigated. Protein expression of adipose triglyceride lipase (ATGL) and perilipin, as well as phosphorylation of hormone-sensitive lipase (HSL), AMP-activated protein kinase (AMPK), and mitogen-activated protein kinases (MAPKs) were determined by western blotting. Moreover, nucleosides, important constituents of PHME, were analyzed using high performance liquid chromatography (HPLC).

**Results:**

Treatment with PHME led to a significant increase in glycerol release thereby reduced intracellular triglyceride accumulation in fully differentiated adipocytes. PHME upregulated protein kinase (PK) A-mediated phosphorylation of HSL at serine residues of 563 and 660. Meanwhile, PHME treatment also upregulated phosphorylation of extracellular signal-regulated kinase (ERK), and downregulated the protein level of perilipin. Pretreatment with the PKA inhibitor, H89, blunted the PHME-induced lipolysis and the phosphorylation of HSL (Ser 563 and 660). Moreover, pretreatment with ERK inhibitor, PD98059, weakened the PHME-caused glycerol release and downregulation of perilipin expression. HPLC analysis indicated there were adenosine, cordycepin, uridine and vernine in PHME.

**Conclusions:**

Our results showed that PHME significantly induced lipolysis in 3T3-L1 adipocytes, which is mainly mediated by activation of HSL through PKA pathway and by downregulation of perilipin through activation of ERK pathway.

**Electronic supplementary material:**

The online version of this article (10.1186/s12906-018-2389-0) contains supplementary material, which is available to authorized users.

## Background

*Cordyceps sinensis* has been used for centuries in China as one of the most valued traditional Chinese medicine and tonic food [1, 2]. In China, it is found in the soil of prairies at elevations of 3500–5000 m, mainly in the provinces of Qinghai, Tibet, Sichuan, Yunnan, and Gansu [3]. It has a wide range of pharmaceutical benefits, such as treatment for kidney and lung disorders, hemostatic and expectorant effects, anti-cancer, immune modulation, hypolipidemic and hypoglycemic activities [4–7]. Because of the limited habitat and over exploitation by humans, *C. sinensis* has been scarce in the nature. Therefore, its usage is limited due to the limited natural resource and high price [8]. Since 1970s, many scientists have been making much efforts to find the alternative material based on fermentation and cultivation of fungal species isolated from natural *C. sinensis* [8]. Among the colonized fungi in *C. sinensis*, *Paecilomyces hepiali* is one of the most important species [9]. The mycelia of this fungus are cultivated by artificial deep fermentation, and have been used widely as a substitute of *C. sinensis* in medicine and health foods, such as ‘JinShuiBao’ capsule, the commercial product of *P*. *hepiali* mycelia, has been used in clinics throughout China [3]. *P. hepiali* has been reported to exhibit anti-oxidation [10], anti-fatigue [11], anti-nociceptive [12], anti-tumor [13], anti-inflammatory [14], anti-diabetic [15] and anti-hyperlipidic effects [3, 15]. The previous works of our Lab found that methanol extract from mycelia of *P. hepiali* (PHME) reduced fat accumulation in adipocyte via suppressing preadipocyte differentiation (Data not shown). These findings suggested this fungus has potential on regulating the metabolism of triglyceride.

It is known that triglyceride (TG) is the major energy storage form in mammals. The major site for storage of TG is the white adipose tissue [16]. In addition as a passive site for storage of energy in the form of TG, adipose tissue is considered as an important endocrine gland which secretes several bioactive molecules [17, 18]. White adipose tissue stores TG during periods of energy excess, and hydrolyzes TG (lipolysis) to release fatty acids (FA) for use by other tissues during times of energy need [19]. However, a dysregulation of lipolysis may lead to metabolic abnormalities. Reduced lipolytic activity may contribute to accumulation of TG in adipose tissue and thus led to obesity, a major risk factor for metabolic disorders including type 2 diabetes and cardiovascular disease [19, 20]. Thus, strategies aimed at increasing lipolysis might be useful in preventing obesity and metabolic diseases [19].

Unlike TG synthesis that occurs in other organs, lipolysis is unique to adipocytes [19]. Adipose lipases perform significant functions in lipolysis because they catalyze the steps of hydrolysis and cleave TGs, diglycerides, and monoglycerides at various stages [21]. Adipose triglyceride lipase (ATGL) and hormone sensitive lipase (HSL) are quantitatively the most important lipases [22]. Activity of HSL is regulated by PKA-catalyzed phosphorylation at serine residues of 563, 659 and 660 [23, 24]. Other kinases, including AMPK, ERK, glycogen synthase kinase-4, and Ca^2+^/calmodulin-dependent kinase, also phosphorylate HSL to modulate its enzyme activity [24]. Besides ATGL and HSL, perilipin A, a lipid droplet-associated protein, has also been considered as the major regulator for lipolysis in adipocytes [25]. Upon hormonal stimulation, perilipin A undergoes phosphorylation by PKA, and then facilitates the translocation of HSL to the lipid droplet and catalyses the lipolysis. However, perilipin A limits lipase access to the lipid droplet, thereby suppressing lipolysis in basal conditions [22, 24]. Therefore, decreased protein expression may impair the barrier function of perilipin A and subsequently lead to the increase in lipolysis. During this process, ERK activation is an early signal for the reduction in perilipin protein expression and subsequent induction of lipolysis [26].

Methanol extract from mycelia of *Paecilomyces hepiali* (PHME) have been shown to decrease TG level in serum and suppress adipogenesis in adipocytes. However, effect of PHME on lipolysis is relatively limited. Whether PHME can modulate HSL or perilipin A to affect lipolysis is still unknown. Therefore, the aim of this study was to clarify the effect of PHME on lipolysis, HSL and perilipin A, and further to elucidate the underlying mechanism in adipocytes.

## Methods

### Chemicals and reagents

Dulbecco’s Modified Eagle Medium (DMEM) was purchased from Life Technologies (Carlsbad, CA). Fetal bovine serum and newborn calf serum were supplied by Bovogen Biological (Melbourne, Australia). Penicillin and streptomycin were purchased from Gibco (New York, NY). 3-isobutyl-1-methylxanthine (IBMX) and indomethacin (Indo) were from Aladdin Inc. (Shanghai, China). Trypsase was purchased from Shanghai Peiyuan biotechnology Co. Ltd. Dexamethasone (DEX), Oil Red O and MTT were supplied by Sigma (St Louis, MO). Bovine serum albumin (BSA) was from MP (Santa Ana, CA).

### Preparation of extract

Dried mycelia of *P. hepiali* were kindly provided by Prof. Yu-quan Xu (Shanghai Jiaotong University, Shanghai, China), and the voucher specimen (No.: Xu Y.Q. 2,015,002) was deposited in HSNU. The *P. hepiali* strains were originally isolated, identified and fermented by Prof. Xu’s laboratory. According to the previous report, methanol extract of *P. hepiali* mycelia was prepared with some modifications [27]. Dried mycelia were crushed and then extracted with 20 volumes of 100% methanol three times (3 h each) at 70 °C under reflux. After filtration, the methanol-extracted solution was concentrated and further dried in vacuum drying oven to produce the methanol extract of *P. hepiali* mycelia (PHME). The PHME was stored in a dark dryer at room temperature.

### Cell culture and differentiation

3T3-L1 mouse embryonic fibroblasts were obtained from the National Center for Drug Screening (Shanghai, China). Preadipocyte differentiation was induced as described previously [28]. Briefly, 3T3-L1 preadipocytes were cultured in high-glucose DMEM supplemented with 10% newborn calf serum and antibiotics (100 μg/mL streptomycin and 100 U/mL penicillin) at 37 °C under a 5% CO_2_ atmosphere. After confluence, 3T3-L1 preadipocytes were maintained in culture medium for two days to induce differentiation (Day 0). Then, cells were incubated in differentiation medium 1 (DM1: DMEM containing 10 μg/mL insulin, 0.5 μM IBMX, 1 μM DEX, 0.2 mM indomethacin, and 10% fetal bovine serum) for three days (Day 3). Then, the medium was changed to differentiation medium 2 (DM2: DMEM containing 10 μg/mL insulin and 10% fetal bovine serum) for additional three days (Day 6). Subsequently, the cells were maintained in DMEM containing 10% fetal bovine serum for another two days (Day 8). Afterward, the fully differentiated adipocytes were harvested for further experiments.

### Cell viability assay

MTT assay was used to evaluate the effect of PHME on cell viability of 3T3-L1 adipocytes [29]. Briefly, 3T3-L1 preadipocytes were seeded in 96 well plates, after differentiation to mature adipocytes (8 days), adipocytes were incubated in DMEM containing 0.2% BSA for 12 h. Afterward, replaced the media with DMEM containing 0.2% BSA and various concentrations of PHME for 48 h. After incubation, 20 μL of 3 mg/mL MTT was added in each well and incubated for 4 h. Then, the culture medium was removed and added 200 μL DMSO in each well. The plate was placed for 20 min at room temperature and then the absorbance was detected at 490 nm on a plate reader (BioTek Inc., VT, USA).

$$ Cellviability\%=\frac{\mathrm{ODsample}-\mathrm{ODblank}}{\mathrm{ODcontrol}-\mathrm{ODblank}}\times 100\% $$

### Oil red O staining

To measure the intercellular lipid accumulation in fully differentiated adipocytes, cells were stained with oil red O as described by Wang [28]. Adipocytes were washed twice with phosphate-buffered saline (PBS, pH 7.4) and fixed with 10% formalin in PBS at room temperature for at least 1 h and then washed with 60% isopropanol. After that, 500 μL of 0.21% (*w*/*v*) oil red O solution (working solution) was added in each well to stain the lipids for 10 min at room temperature. Freshly diluted oil red O working solution contained six parts of oil red O stock solution (0.35% oil red O in isopropanol) and four parts of H_2_O. Cells were washed with distilled water four times and then added with 100% isopropanol for 10 min to dissolve the bound staining. Afterward, the absorbance was measured at 500 nm.

### Lipolysis measurement

Lipolysis was evaluated by measuring the amount of glycerol released into the medium [28]. After starvation, differentiated 3T3-L1 adipocytes were treated with PHME (100, 200, 300, 400 and 500 μg/mL) for 24, 48 and 72 h. Then the supernatant of the media was collected for glycerol release measurement using a glycerol assay kit (APPLYGEN Beijing, China).

### Protein extraction and Western blotting analysis

Fully differentiated 3T3-L1 cells treated or untreated with PHME were washed twice with cold PBS buffer and then lysed in a lysis buffer (150 mM sodium chloride, 1.0% Triton X-100, 0.5% sodium deoxycholate, 0.1% sodium dodecyl sulfate (SDS), 50 mM Tris, pH 8.0) containing protease and phosphatase inhibitors. Cell lysates were incubated in ice for 30 min, vortexed every 5 min, lysed at − 80 °C overnight, and then were centrifuged at 12,000 rpm for 20 min at 4 °C [28]. The protein concentrations of the supernatant were determined by BCA (bicinchonininc acid) protein assay kit (YEASEN Biotechnology Co., Ltd., Shanghai, China) using BSA (bovine serum albumin) as standard. Proteins (50 μg per lane) were subjected to electrophoresis and separated by 12% SDS- polyacrylamide gel electrophoresis and transferred to nitrocellulose membranes (ExCell, China). The membranes were blocked in 5% non-fat drymilk solution at room temperature for 1 h and incubated with the primary antibodies at 4 °C for overnight. Then the membranes were washed with TBST (Tris Buffered saline Tween) and then incubated with the secondary antibodies (Gaithersburg, MD; 1:10000) for 1 h at room temperature. Images were captured using the Odyssey CLx Imaging System (LI-COR, Lincoln, NE) [28].

### HPLC analysis

#### Sample preparation

A stock solution of 2 mg/mL of PHME was made in 15% methanol. The solution was ultrasonicated for 5 mins and filtered through a 0.22 μm membrane filter before HPLC analysis.

#### Chromatographic conditions

HPLC analysis was performed on an Agilent 1100 Series liquid chromatograph system and an ultraviolet detector (Agilent Technologies, Palo Alto, California, USA). Based on previous reports [2, 8, 30] and preliminary studies, the wavelength was selected 260 nm for uridine, vernine and adenosine, and selected 254 nm for cordycepin analysis. Separation was conducted on a C18 analytical column (250 mm × 4.6 mm, No.LAAI-KR006, Kromasil) at 30 °C with a flow rate of 1.0 mL/min and injection volume of 10 μL. In analysis of uridine, vernine and adenosine, the mobile phase consisted of two solvents (*v*/v): 85% phosphate buffer (Na_2_HPO_4_-NaH_2_PO_4_, pH 6.5) and 15% methanol. Meanwhile, the mobile phase was 20% methanol solution for cordycepin analysis.

#### Calibration curves

Four standard samples, uridine (100 μg/mL), vernine (50 μg/mL), adenosine (80 μg/mL) and cordycepin (50 μg/mL) were prepared by dissolving in 15% methanol, respectively. Then stock solutions were diluted with 15% methanol to appropriate concentrations for establishing calibration curves. The calibration curves were constructed by plotting the relative peak areas vs. the concentrations of each standard sample.

#### Precision

In order to detect the precision and stability of the HPLC system, the precision test, stability test and sample recovery test were executed, respectively. Certain concentrations of standard and sample solutions were tested. Every concentration was tested for at least five times. Variations were expressed by relative standard deviations (R.S.D.).

### Statistical analysis

The data were presented as mean ± SD and analyzed by one-way ANOVA followed by Duncan’s multiple range test using SPSS software (13.0 ed.). *P* < 0.05 and *P* < 0.01 were considered statistically significant and extremely significant between groups, respectively.

## Results

### Effects of PHME on cell viability of 3T3-L1 adipocytes

**Fig. 1:**
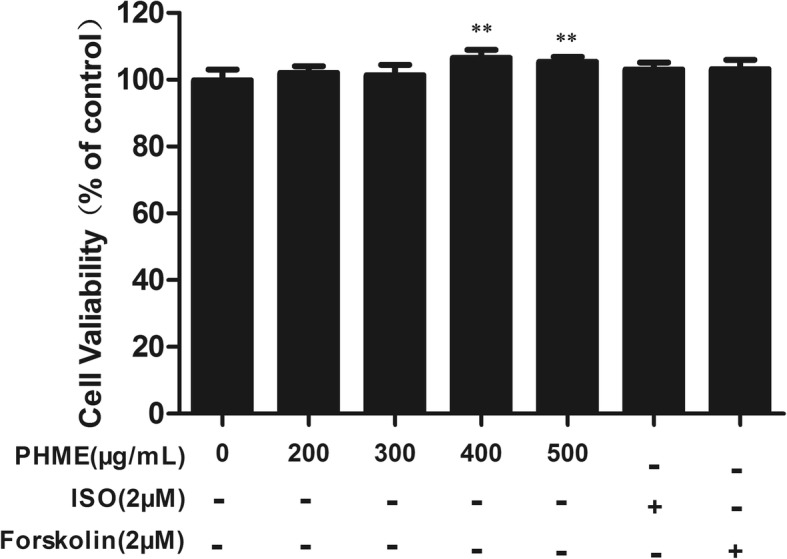
Effect of PHME on viability of fully differentiated 3T3-L1 adipocytes. The cell viability was analyzed by MTT assay after treatment of PHME (200–500 μg/mL) for 24 h, and cells treated with 0.1% dimethyl sulfoxide were used as control group. Isoproterenol (ISO) and forskolin were used as positive controls. Data represent as means±SD (*n* = 6), **P < 0.01 vs. control group

The potential cytotoxicity of PHME on fully differentiated 3 T3-L1 adipocytes was evaluated by MTT assay. As results shown in Fig. 1, 200 and 300 μg/mL PHME didn’t affect the viability of 3T3-L1 adipocytes (*P* > 0.05). Treatment with 400 and 500 μg/mL PHME for 24 h increased the cell viability compared with control cells by 6.5 and 5.3%, respectively (*P* < 0.01). Our data showed that PHME had no cytotoxic effect against fully differentiated 3T3-L1 adipocytes. Therefore, PHME at concentrations less than 500 μg/mL were used in the following study.

### PHME stimulated basal lipolysis in 3T3-L1 adipocytes

**Fig. 2:**
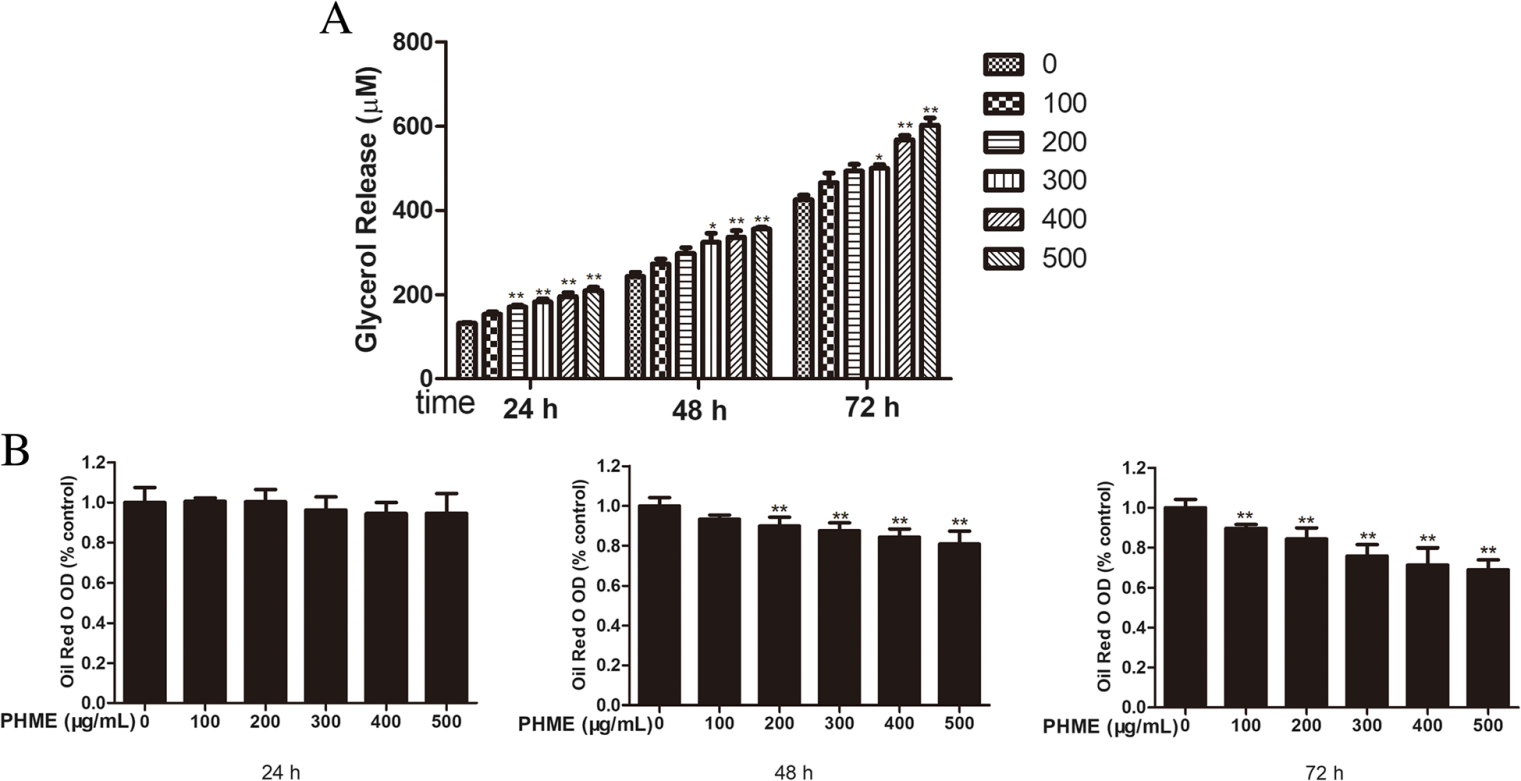
Effect of PHME on adipocyte lipolysis. **a** Fully differentiated adipocytes treated with various concentrations of PHME (100, 200, 300, 400 and 500 μg/mL) for 24, 48 and 72 h, respectively. Then, glycerol contents in media were measured by glycerol assay kit. **b** Adipocytes treated with PHME for 24, 48 and 72 h were stained with oil red O and quantified intracellular lipid by measuring the absorbance at 500 nm. Values are expressed as mean ± SD (*n* = 4), *P < 0.05 and **P < 0.01 vs. control group, respectively

Triglyceride hydrolysis releases glycerol and free fatty acids from adipocytes. Contents of glycerol released into the medium and intracellular triglyceride were assayed as indices of lipolysis. Fully differentiated 3T3-L1 adipocytes were incubated with various concentrations of PHME for 24, 48 and 72 h. As results shown in Fig. 2a, 200, 300, 400 and 500 μg/mL PHME significantly increased glycerol contents in the medium (*P* < 0.05 or *P* < 0.01), whereas 100 μg/mL PHME hardly exhibited any effect on glycerol release (*P* > 0.05). Oil red O staining was performed to confirm lipolytic effect of PHME, and results (Fig. 2b) showed that all concentrations of PHME significantly decreased the content of intracellular triglyceride after 72 h of treatment (*P* < 0.01). These results revealed PHME significantly stimulated basal lipolysis in mature 3T3-L1 adipocytes.

### Effects of PHME on the main lipases and kinases involved in lipolysis regulation

**Fig. 3:**
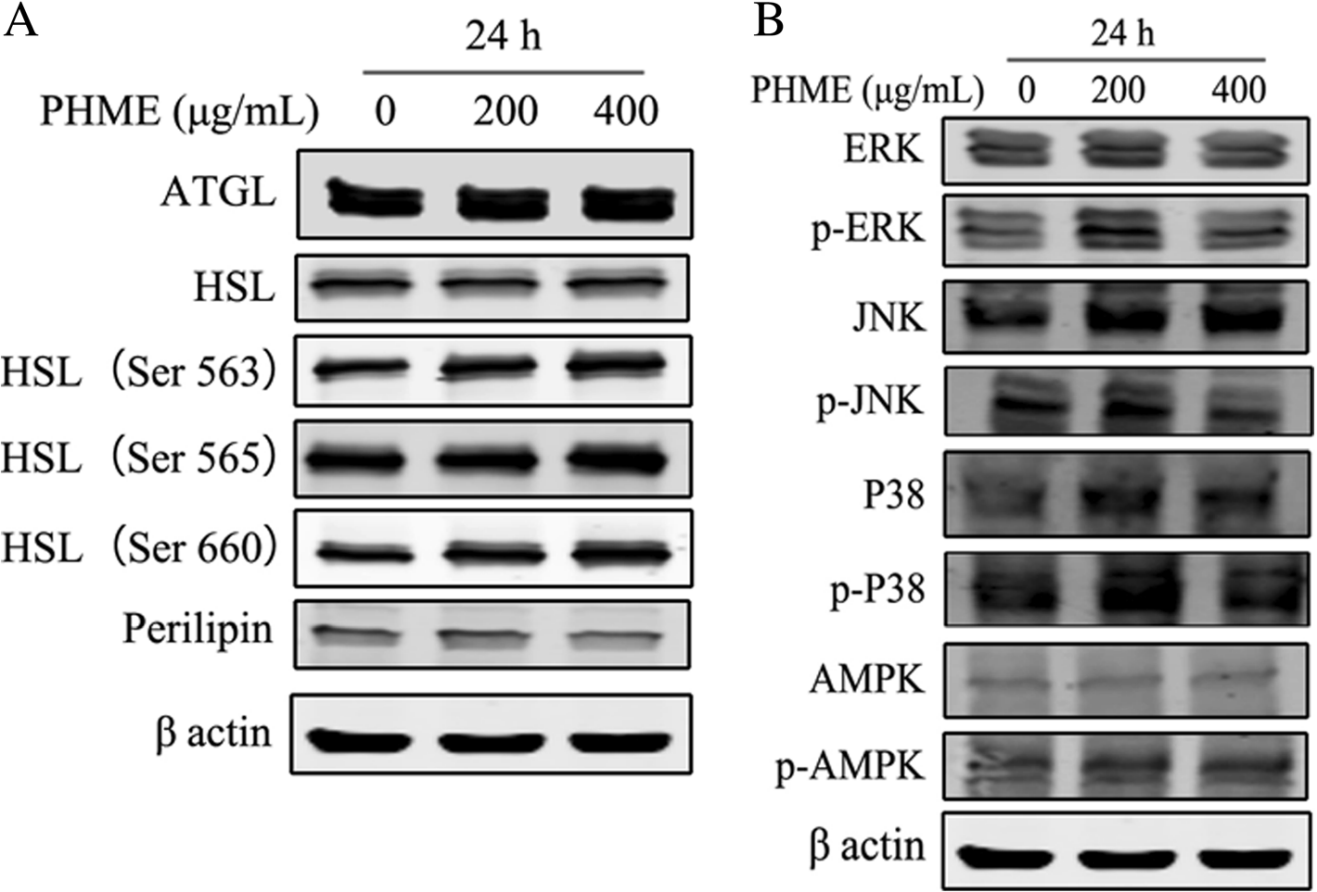
Effect of PHME on the expression of ATGL, perilipin, HSL and phosphorylated HSL at Ser563, Ser565 and Ser660 (**a**), and on the expression and phosphorylation of ERK, p38, JNK and AMPK (**b**). Fully differentiated 3 T3-L1 adipocytes were treated with 200 and 400 μg/mL PHME for 24 h, then expression of ATGL and perilipin, as well as basal and phosphorylated levels of HSL, AMPK and MAPKs were measured using western blotting analysis

ATGL, HSL and perilipin have been reported as the major regulators for lipolysis control. To investigate the underlying molecular mechanisms by which PHME stimulates adipocyte lipolysis, we performed western blotting analysis to examine the involvement of these regulators. As results shown in Fig. 3a, PHME treatment (200 and 400 μg/mL) did not modify protein expression of ATGL levels, but it increased PKA-dependent phosphorylation of HSL at Ser563 and Ser660. Moreover, the protein expression of perilipin was markedly downregulated by PHME treatment (Fig. 3a).

AMP-activated protein kinase (AMPK) and mitogen-activated protein kinases (MAPKs) signaling pathways were involved in the regulation of lipolysis. In this study, we tested the phosphorylation of AMPK and three MAPKs. As shown in Fig. 3a and b, PHME treatment did not affect phosphorylation of AMPK and AMPK-mediated phosphorylation of HSL at Ser565. After 24 h treatment, PHME upregulated the phosphorylation of ERK, but it did not modify the phosphorylation of JNK and p38 (Fig. 3b). These results suggested that PKA and ERK signaling pathways were probably involved in PHME-promoted adipocyte lipolysis.

### PHME increased HSL phosphorylation through activation of PKA pathway

**Fig. 4:**
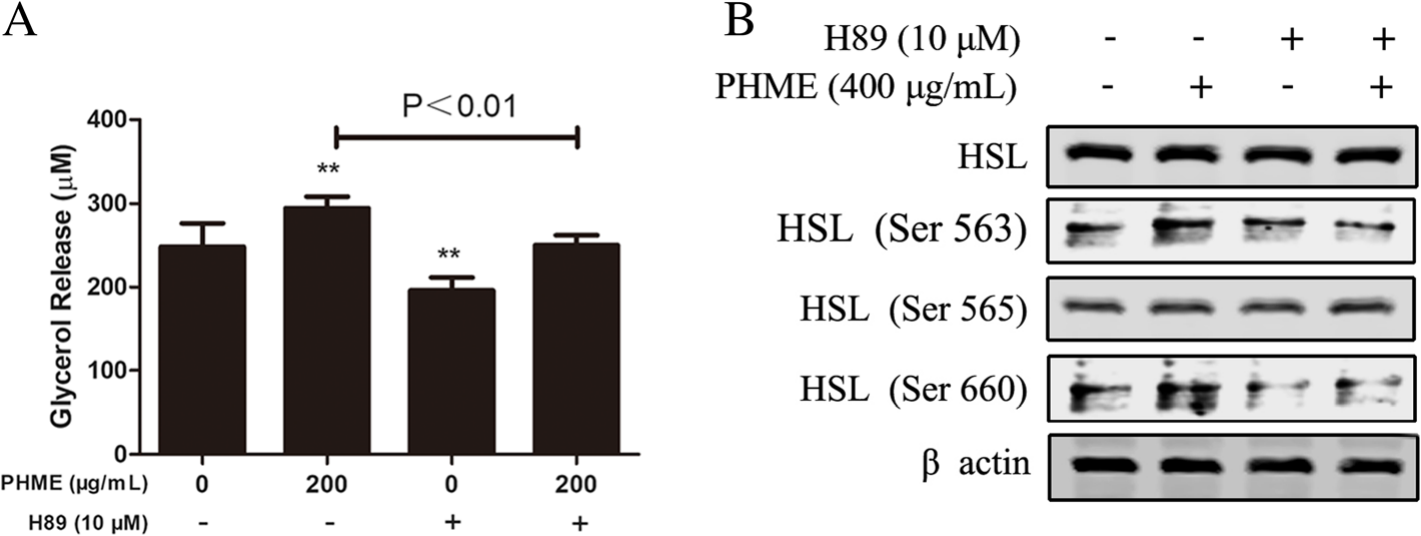
Effects of a PKA inhibitor (H89) on PHME-stimulated lipolysis. **a** Fully differentiated 3T3-L1 adipocytes were treated with 200 μg/mL PHME alone or in the presence of 10 μM H89 for 12 h, then glycerol released into the medium was quantified. **b** Fully differentiated 3T3-L1 adipocytes were treated with 400 μg/mL PHME alone or in the presence of 10 μM H89 for 12 h, then cells were lysed, and then basal and phosphorylated levels of HSL were measured using western blotting analysis. Values are expressed as mean ± SD (*n* = 4), **P < 0.01 vs. control group

To further investigate whether PKA pathway was involved in mediating PHME-stimulated lipolysis, fully differentiated 3T3-L1 adipocytes were treated with PHME for 12 h with or without H89 (PKA inhibitor), and then glycerol release were measured. As glycerol contents showed in Fig. 4a, 200 μg/mL PHME significantly promoted content of glycerol released into the medium (*P* < 0.01 vs. control group), and this effect was abolished by pretreatment with H89 (*P* < 0.01 vs. PHME-treated alone). Furthermore, the blocking of PKA also reversed PHME-induced upregulated phosphorylation of HSL at residues of Ser563 and Ser660 (Fig. 4b). These results indicated that PHME upregulated HSL phosphorylation through activation of PKA pathway, supporting the key role of the PKA pathway in lipolytic action of PHME.

### PHME downregulated the protein expression of perilipin through ERK activation

**Fig. 5:**
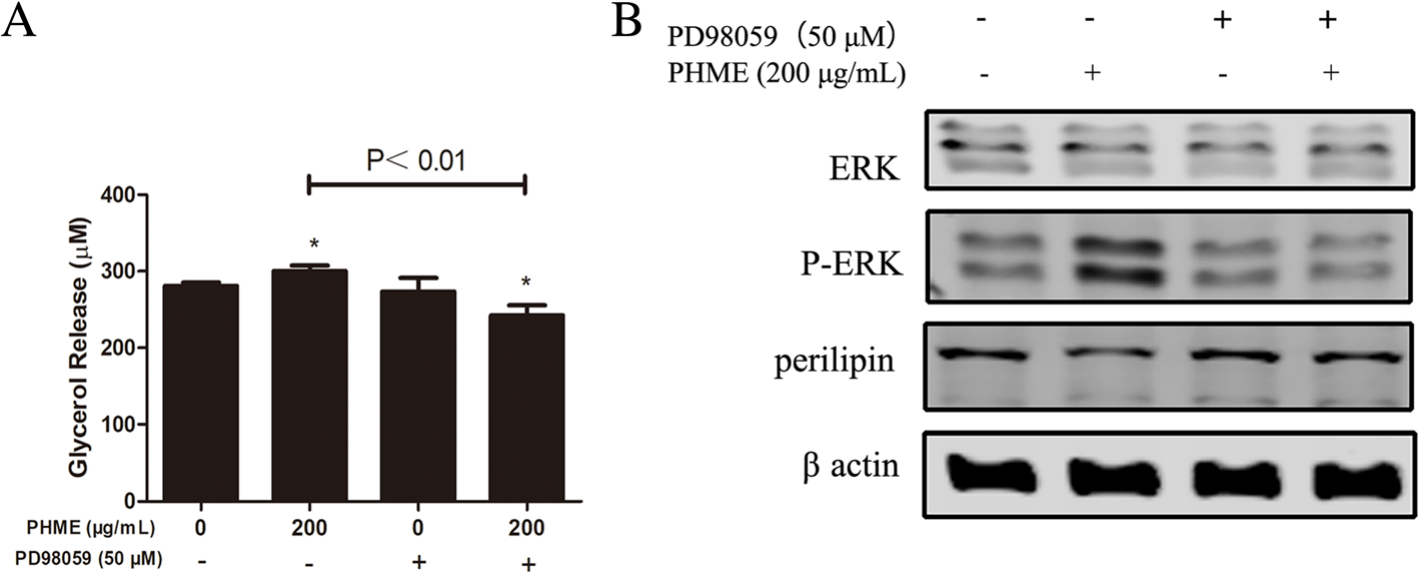
Effects of an ERK inhibitor (PD98059) on PHME-stimulated lipolysis. **a** Fully differentiated 3T3-L1 adipocytes were treated with 200 μg/mL PHME alone or in the presence of 50 μM PD98059 for 12 h, then contents of glycerol released into the medium were quantified. **b** Fully differentiated 3T3-L1 adipocytes were treated with 200 μg/mL PHME alone or in the presence of 50 μM PD98059 for 20 h, then cells were lysed, and then ERK phosphorylation and expression of perilipin were measured using western blotting analysis. Values are expressed as mean ± SD (*n* = 4), *P < 0.05 vs. control group

To investigate the possible role of the ERK pathway in PHME-stimulated lipolysis, an ERK inhibitor (PD98059) was employed to block activation of the ERK pathway. First, the effect of PD98059 on PHME-stimulated adipocyte lipolysis was evaluated. As results showed in Fig. 5a, after treatment for 12 h, PHME significantly promote glycerol release in the culture (*P* < 0.05 vs. control group). Pretreatment with PD98059 attenuated PHME-caused glycerol release (*P* < 0.01 vs. PHME-treated group). Furthermore, PHME treatment decreased the protein expression of perilipin, and this phenomenon was also abolished by PD98059 treatment (Fig. 5b). The results suggested that PHME stimulated lipolysis by decreasing perilipin via activation of ERK pathway.

### HPLC analysis of PHME

HPLC analysis of PHME revealed four peaks matching commercial standards: uridine, vernine, adenosine, and cordycepin, with retention times of approximately 4.14, 5.56, 11.36 and 4.41 min, respectively (Additional file 1: Figure S1).

Quantification of nucleosides was analyzed using standard curves. According to the UV spectra of analytes, 260 nm was used as detection wavelength for uridine, vernine, and adenosine, as well as 254 nm was used as detection wavelength for cordycepin. As shown in Additional file 2: Table S1, a satisfactory linear relationship was obtained between the concentrations of the analyte and corresponding peak area. The precision results demonstrated that the developed method is precise and sensitive for the quantitative determination of uridine, vernine, adenosine, and cordycepin in PHME. Moreover, the calibration curves of four analytes were calculated by plotting the peak areas (y) vs. the corresponding concentrations (x, μg/mL) by using the standard solutions (Additional file 2: Table S1). The amount of four analytes were calculated by calibration curves listed in Additional file 2: Table S1, respectively. The contents of uridine, vernine, adenosine and cordycepin in PHME were 0.94 ± 0.002, 0.44 ± 0.001, 0.33 ± 0.001 and 0.53% ± 0.001 (*n* = 5), respectively.

## Discussion

**Fig. 6:**
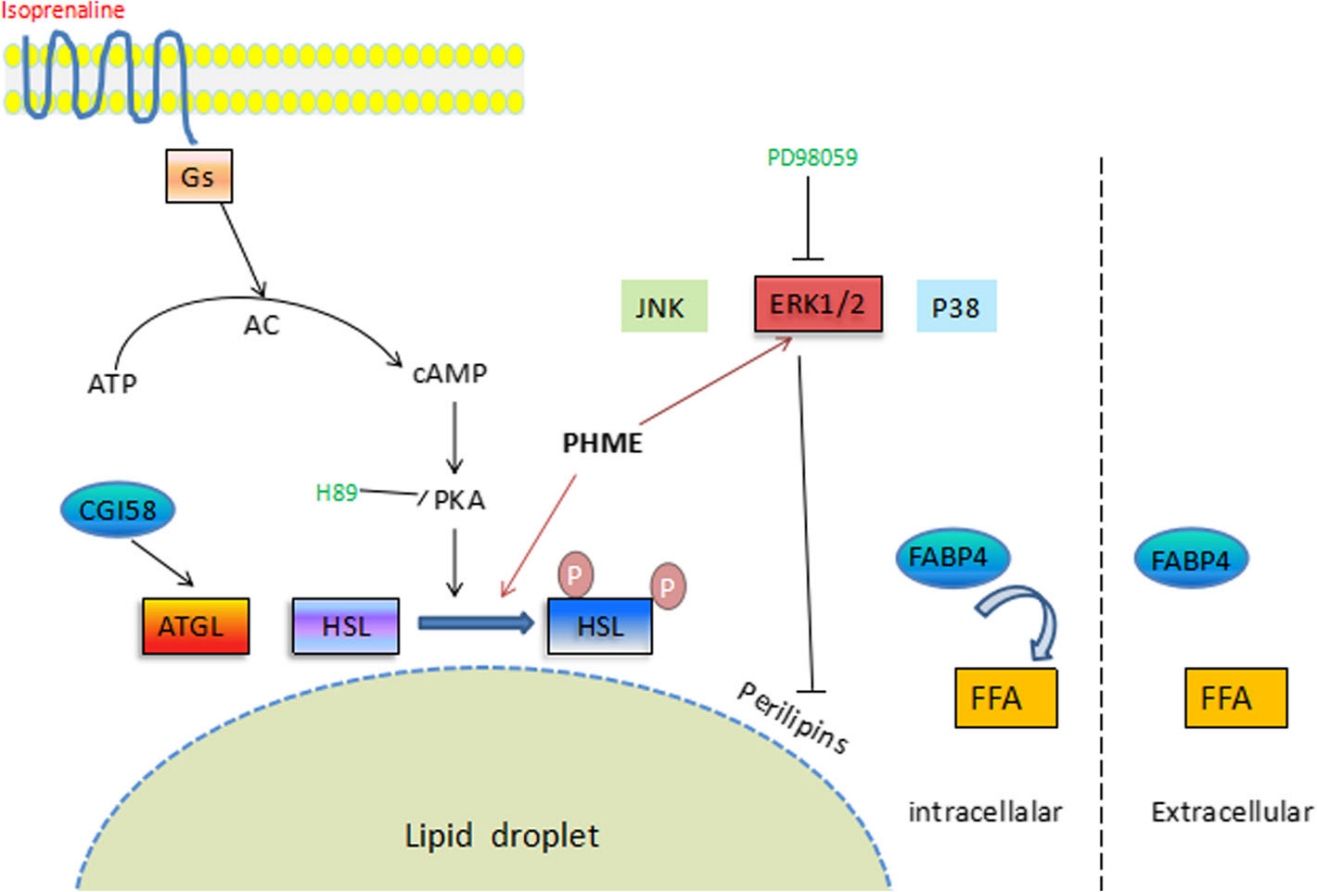
Mechanisms for the lipolytic effect of PHME in 3T3-L1 adipocytes. PHME stimulates lipolysis via activating HSL and downregulating perilipin through PKA and ERK signaling pathways

In the current study, we found PHME exhibited lipolytic activity in 3T3-L1 adipocytes. This extract significantly stimulated adipocyte lipolysis and reduced intracellular triglyceride accumulation through activation of HSL and downregulation of perilipin (Fig. 6). PKA and ERK signal pathways were involved in mediating the lipolytic effect of PHME.

In this study, we found that PHME increased phosphorylation of HSL at Ser563 and Ser660. It is well-known that phosphorylation of HSL at Ser563, Ser659 and Ser660 by PKA activates adipocyte lipolysis [22]. PKA phosphorylates HSL resulting in increased hydrolytic activity, translocation of HSL from cytosol to the lipid droplet surface, and hydrolyzed DAG [31]. Our results suggested that PHME enhanced glycerol release probably through activation of PKA pathway. Then, an inhibitor of PKA, H89, was used to confirm this hypothesis and found H89 blunted the PHME caused phosphorylation of HSL at Ser563 and Ser660 (major PKA-targets), and the subsequent increase in glycerol release. These findings revealed that PHME -stimulated lipolysis was dependent on PKA activation.

Moreover, we observed that PHME treatment caused a significant decrease in protein expression of perilipin A. Perilipin A plays an essential role in the control of lipolysis [25]. In basal condition, perilipin A acts as a barrier to lipases, thereby maintaining a low rate of basal lipolysis [22]. Thus, a decrease in perilipin A expression allows access of the lipases to the lipid droplet and enhanced lipolysis [25]. The ERK signaling pathway is involved in regulating perilipin protein expression and perilipin phosphorylation. ERK activation is able to regulate adipocyte lipolysis by phosphorylating HSL at Ser600 and therefore increasing the activity of HSL [32]. In addition, ERK activation is reported as an early signal for the reduction in perilipin protein expression and subsequent induction of lipolysis [24]. Previous studies revealed that TNF-α stimulated lipolysis in 3T3-L1 and human adipocytes are dependent on down-regulation of perilipin expression via activation of ERK pathway [26, 33].

In the present study, incubation with PHME resulted in a significant increase in lipolysis, which were paralleled by a decrease in the protein expression of perilipin A and an increase in level of phosphorylated ERK. It is known that decreased protein expression may impair the barrier function of perilipin A and thus lead to the increased lipolysis. Thus, we speculate that downregulating of perilipin A expression caused by PHME is one of its lipolytic mechanisms, and ERK pathway probably be involved in regulation of perilipin A expression. To test this possibility, we used PD98059 (an inhibitor of ERK) to block the ERK activity during 3T3-L1 adipocyte lipolysis and found that both PHME-caused lipolysis and downregulation of perilipin A expression were inhibited in the presence of PD98059. Therefore, these results indicated that PHME-caused lipolysis is dependent on down-regulation of perilipin expression via ERK pathway.

AMPK pathway also involves in the regulation of lipolysis. Indeed, translocation of HSL to the lipid droplets inhibited by AMPK through phosphorylation at Ser565 is well established [25]. Our current data showed that phosphorylation of HSL at Ser565 and phosphorylated AMPK level weren’t affected by PHME treatment. These results indicated that AMPK pathway wasn’t involved in mediating the lipolytic effect of PHME.

Nucleosides, one of the major ingredients in *Cordyceps*, are believed to be the active components in *Cordyceps* and used as marker for quality control of *Cordyceps* [2, 3]. Such as adenosine is a marker for quality control of *C. sinensis* [2]. As the important fungal species isolated from natural *C. sinensis, Paecilomyces hepiali* also contain bioactive nucleosides which used as marker for quality control. It is known the amount and sources of nucleosides in natural *Cordyceps* are different from that of cultured one [2]. Moreover, the content and sources of nucleosides in cultured mycelia are affected by submerged culture conditions and nutritional requirements. Therefore, main constituents of nucleosides were analyzed in the present study. Results of HPLC showed there were four nucleosides including adenosine, cordycepin, uridine and vernine in PHME. In order to clarify the active compound, we further evaluated effect of these four nucleosides on adipocyte lipolysis, and found only cordycepin exhibited significant lipolytic action (Additional file 3: Figure S2). Cordycepin were reported to possess anti-cancer, anti-oxidant and immunomodulating activities [34]. Moreover, cordycepin suppressed differentiation of 3T3-L1 pre-adipocytes and pre-adipocytes in primary culture, and this anti-adipogenic effect occur through its intervention in the mTORC1-C/EBPβ–PPARγ pathway [35]. Therefore, it is suggested that cordycepin might be one of the main active constituents in PHME that could enhance lipolysis via the PKA and ERK signaling pathways. However, further studies would be crucial to investigate mechanism of lipolytic effect of cordycepin.

## Conclusions

In summary, the present data demonstrate that methanol extract from mycelia of *Paecilomyces hepiali* induced HSL phosphorylation and decreased perilipin level, therefore increased lipolysis in fully differentiated 3T3-L1 adipocytes. PHME-induced lipolysis was mediated via at least two different pathways. One involved PKA activation, leading to phosphorylation of HSL at Ser 563 and 660 to hydrolyze triacylglycerols in adipocytes. In the other pathway, PHME induced the phosphorylation of ERK, thereby decreasing the levels of perilipin to promote lipolysis. *P. hepiali* has been widely used as a medicine or a dietary supplement. Our results provided a potential mechanism by which *P. hepiali* could improve lipid disorders.

## Additional files

Additional file 1: **Figure S1.** HPLC analysis of PHME. (A) HPLC chromatogram of PHME at 260 nm. (B) HPLC chromatogram of PHME at 254 nm. Uridine (1), vernine (2), adeosine (3), and cordycepin (4) were determined, respectively. (TIF 459 kb)

Additional file 2: **Table S1.** Linear regression data and precision of four nucleosides (DOCX 17 kb)

Additional file 3: **Figure S2.** Effects of adenosine, vernine, uridine, mannitol and cordycepin on lipolysis in 3T3-L1 adipocytes. Mature 3T3-L1 adipocytes were treated with 10–100 μmol/L Adenosine (A), vernine (B), uridine (C), mannitol (D) for 24 h, and 10–100 μmol/L cordycepin (E) for 24, 48, and 72 h, then the amounts of glycerol released into the culture medium were measured. Values are expressed as mean ± SD (*n* = 4), **P < 0.01 vs. control group. (TIF 419 kb)

## Abbreviations

AMPK: AMP-activated protein kinase
ATGL: Adipose triglyceride lipase
ERK: Extracellular signal-regulated kinase
FA: Fatty acids
HPLC: High performance liquid chromatography
HSL: Hormone-sensitive lipase
MAPK: Mitogen-activated protein kinase
PHME: Methanol extract of *Paecilomyces hepiali* mycelia
PKA: Protein kinase A
TG: Triglyceride
WAT: White adipose tissue
