# Supplementary figures and images for: Extract of Paecilomyces hepiali mycelia induces lipolysis through PKA-mediated phosphorylation of hormone-sensitive lipase and ERK-mediated downregulation of perilipin in 3T3-L1 adipocytes
Source: BMC Complement Altern Med. 2018 Dec 7;18:326. doi: 10.1186/s12906-018-2389-0 (PMC6286538; doi:10.1186/s12906-018-2389-0)

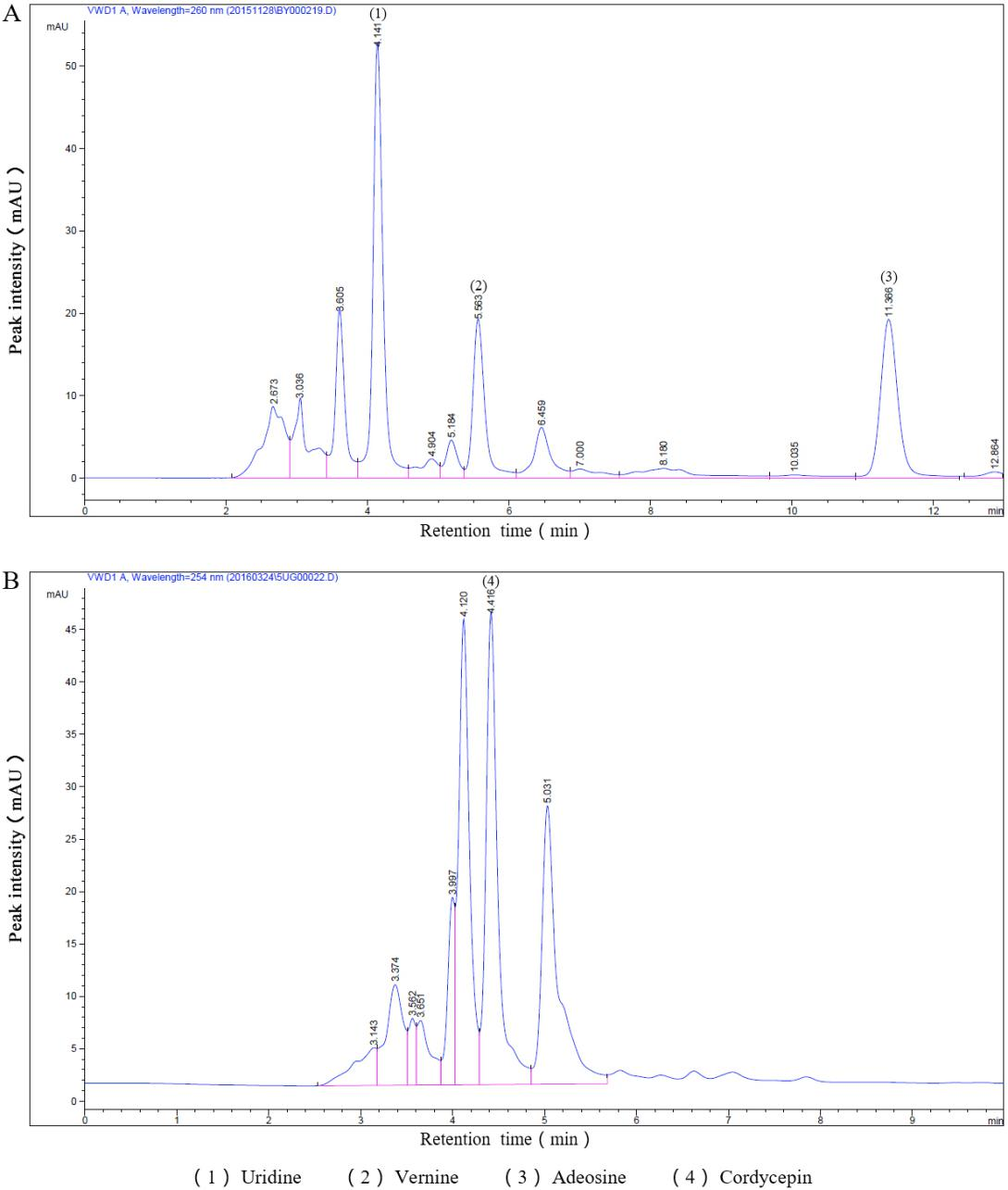

Supplement: Supplementary file 1 — Figure S1. HPLC analysis of PHME. (A) HPLC chromatogram of PHME at 260 nm. (B) HPLC chromatogram of PHME at 254 nm. Uridine (1), vernine (2), adeosine (3), and cordycepin (4) were determined, respectively. (TIF 459 kb) [file 12906_2018_2389_MOESM1_ESM.tif]

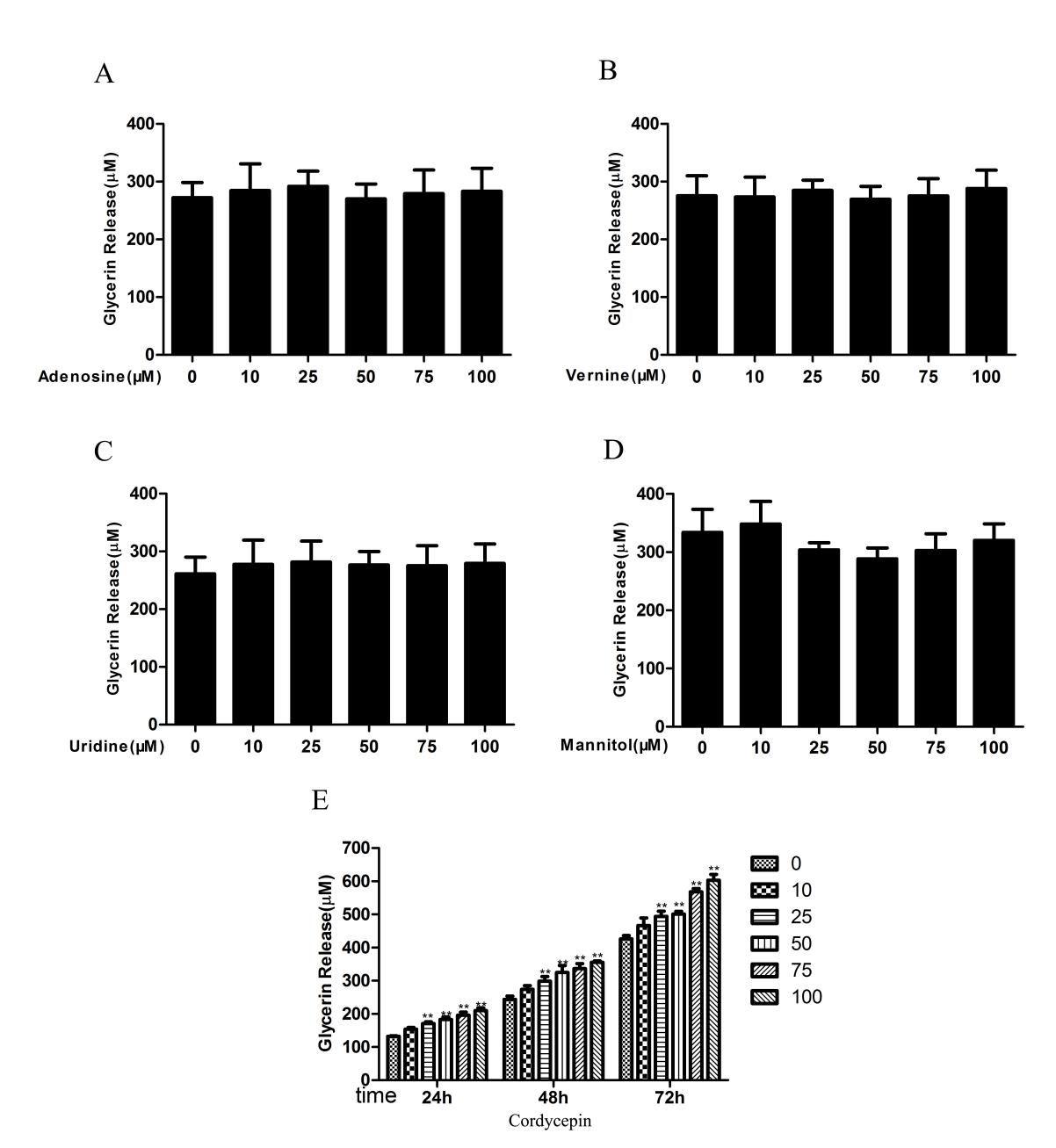

Supplement: Supplementary file 3 — Figure S2. Effects of adenosine, vernine, uridine, mannitol and cordycepin on lipolysis in 3T3-L1 adipocytes. Mature 3T3-L1 adipocytes were treated with 10–100 μmol/L Adenosine (A), vernine (B), uridine (C), mannitol (D) for 24 h, and 10–100 μmol/L cordycepin (E) for 24, 48, and 72 h, then the amounts of glycerol released into the culture medium were measured. Values are expressed as mean ± SD (n = 4), **P < 0.01 vs. control group. (TIF 419 kb) [file 12906_2018_2389_MOESM3_ESM.tif]
