# Supplementary material for: Extract of Paecilomyces hepiali mycelia induces lipolysis through PKA-mediated phosphorylation of hormone-sensitive lipase and ERK-mediated downregulation of perilipin in 3T3-L1 adipocytes
Source: BMC Complement Altern Med. 2018 Dec 7;18:326. doi: 10.1186/s12906-018-2389-0 (PMC6286538; doi:10.1186/s12906-018-2389-0)
Supplement: Supplementary file 2 — Table S1. Linear regression data and precision of four nucleosides (DOCX 17 kb) [file 12906_2018_2389_MOESM2_ESM.docx]

Table S1. Linear regression data and precision of four nucleosides

| Analytes | Linear regression | | | Precision | | |
| --- | --- | --- | --- | --- | --- | --- |
|  | Calibration curves | Correlation coefficient | Linear range(μg/mL) | Precisition test  R.S.D.(%) | Stailiable test  R.S.D.(%) | Sample recovery rate R.S.D.(%) |
| uridine | y=54.292x+20.169 | 0.9996 | 1.5625-100 | 1.15 | 0.98 | 1.43 |
| vernine | y=49.023x-1.7701 | 1.0000 | 1.5625-50 | 0.44 | 1.08 | 1.16 |
| adenosine | y=44.718x+4.8301 | 0.9999 | 1.25-80 | 0.98 | 1.30 | 1.45 |
| cordycepin | y=36.025x+1.2204 | 1.0000 | 0.39-50 | 1.56 | 2.16 | 2.17 |

y and x stand for the peak area (mAU) and the concentration (μg/mL) of the analytes, respectively.
